# Effect of Citric Acid and Tromethamine on the Stability of Eyedrops Containing Lifitegrast

**DOI:** 10.3390/ph17111415

**Published:** 2024-10-23

**Authors:** Ji-Su Jeong, Eun-Sol Ha, Heejun Park, Seon-Kwang Lee, Hui-Taek Kang, Min-Soo Kim

**Affiliations:** 1 College of Pharmacy, Pusan National University, 63 Busandaehak-ro, Geumjeong-gu, Busan 46241, Republic of Korea; sui15@pusan.ac.kr (J.-S.J.); edel@pusan.ac.kr (E.-S.H.); lsk7079@pusan.ac.kr (S.-K.L.); gms1406@pusan.ac.kr (H.-T.K.); 2 College of Pharmacy, Duksung Women’s University, 33, Samyangro 144-gil, Dobong-gu, Seoul 01369, Republic of Korea; heejunpark@duksung.ac.kr

**Keywords:** lifitegrast, dry eye disease, eyedrop formulation, physicochemical stability

## Abstract

Background/Objectives: Lifitegrast is an effective treatment for dry eye disease, reducing inflammation and improving the ocular surface condition. Owing to its high sensitivity to oxidation and hydrolysis, formulation studies are required to maintain the physicochemical stability of lifitegrast. This study aimed to overcome the instability of lifitegrast by developing a more stable eyedrop formulation by using citric acid and tromethamine to prevent the degradation of lifitegrast. Methods: Based on the Design of Experiment (DoE) approach, formulations were prepared at various concentrations of two stabilizers, citric acid and tromethamine. The stabilizers were carefully controlled to reduce the generation of degradation products. The eyedrops were stored under accelerated test conditions, and parameters such as appearance, pH, drug content, and impurities were evaluated. Results: The results showed that all critical quality attributes (CQAs) including appearance, pH, drug content, and impurities were maintained at stable levels under accelerated conditions, meeting established criteria. In addition, it was suggested that citric acid provided protection against oxidative stress, while tromethamine prevented hydrolysis caused by pH fluctuations. Conclusions: Consequently, it was concluded that the developed lifitegrast-containing eyedrop formulation exhibited improved physicochemical stability, validated through statistical analyses. These findings contribute to the development of stable eyedrops and provide a foundation for commercial production and clinical applications.

## 1. Introduction

Dry eye disease (DED) is a complex ocular disorder caused by tear film instability due to insufficient tear production or excessive evaporation [1,2,3]. It is accompanied by inflammation of the ocular surface, leading to discomfort, blurred vision, and eye pain, which severely affect the patient’s quality of life. Over the years, various drugs have been studied and used for the treatment of DED. Among them, cyclosporine A (CsA; Restasis^®^), approved in 2003, has been one of the most widely used therapies due to its ability to reduce ocular surface inflammation by inhibiting T-cell activation. However, CsA has limitations in efficacy and stability, requiring suspension-based formulations due to poor solubility, which can lead to discomfort during administration and storage stability issues. Additionally, CsA has a slower onset of action and is not consistently effective in addressing both the signs and symptoms of DED. In contrast, lifitegrast is soluble in aqueous media, allowing for a clear solution that improves the stability of formulation, and offers a more comprehensive and convenient treatment. Lifitegrast inhibits the interaction between lymphocyte function-associated antigen-1 (LFA-1) and intercellular adhesion mol-ecule-1 (ICAM-1), preventing T-cell-mediated inflammation. Reducing inflammation on the ocular surface helps restore the tear film. Lifitegrast, a crucial drug for treating DED, was approved by the U.S. Food and Drug Administration in 2016, and the commercially available Xiidra^®^, containing 5% lifitegrast, is widely used for the effective treatment of DED symptoms [4,5,6,7,8].

**Figure 1:**
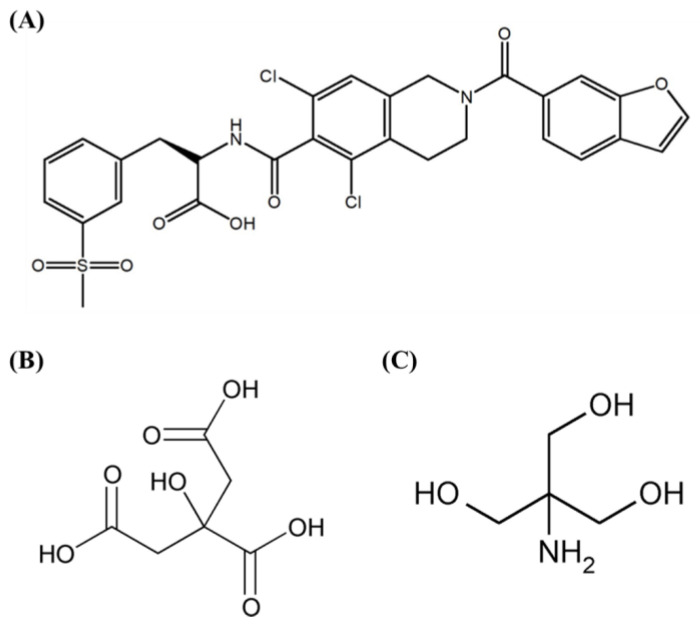
Molecular structures of (**A**) lifitegrast, (**B**) citric acid, and (**C**) tromethamine.

Although lifitegrast provides a better therapeutic profile and improved formulation characteristics in treating dry eye disease, challenges related to its chemical stability still exist. The chemical structure (Figure 1A), which includes an amide bond and a carbonyl group, can undergo hydrolysis in extremely acidic or basic conditions, leading to the formation of degradation products [9]. In addition, oxidizing agents such as reactive oxygen species (ROS) or free radicals convert lifitegrast into N-hydroxy compounds, which are subsequently transformed into iminium ions, thereby significantly contributing to the degradation process through oxidative stress [10,11]. The degradation of lifitegrast diminishes its therapeutic efficacy and can lead to safety risks. To overcome these challenges in eyedrops containing lifitegrast, stabilizers such as antioxidants and pH buffers are essential to maintain drug stability and prevent degradation caused by hydrolysis and oxidation [9,11]. Citric acid, a potent antioxidant, removes ROS, such as hydrogen peroxide (H_2_O_2_), and chelates metal ions to prevent metal-catalyzed oxidative reactions, thereby enhancing the chemical stability of the formulations. The chemical structure of citric acid is shown in Figure 1B [12,13,14]. Tromethamine, as shown by its chemical structure in Figure 1C, acts as a pH buffer, maintaining stability within the pH of 7–9 to prevent the hydrolysis of pH-sensitive drugs, such as lifitegrast, thus reducing degradation [14,15,16].

In this study, the quality target product profile (QTPP) was established to ensure that the formulation met the necessary criteria for efficacy and stability (Table 1). The critical quality attributes (CQA) included maintaining a clear appearance, pH range of 6.8–8.0, drug content between 90.0% and 110.0%, and impurity levels below 1.0%. These critical attributes were identified as essential to guarantee the long-term stability, safety, and therapeutic effectiveness of the product during both development and storage. The critical material attributes (CMA) for the eyedrop formulation were the concentrations of citric acid and tromethamine. Citric acid and tromethamine were chosen as stabilizers to improve the stability of lifitegrast eyedrops by mitigating the degradation caused by oxidation and pH fluctuations. The stabilizer concentrations were adjusted to ensure sufficient stability of the eyedrops using a Design of Experiment (DoE) approach. Citric acid, as an antioxidant, reduced oxidative degradation, while tromethamine acted as a pH buffer to prevent hydrolysis, and their effects on maintaining the critical quality attributes of the eyedrops were confirmed. The critical process parameters (CPP), such as mixing speed, time, temperature, and the order of addition of stabilizers, had minimal impact on the formulation due to the high solubility of the excipients in water [17,18,19,20]. This study is expected to provide practical benefits by enhancing the stability of formulations, improving patient treatment outcomes, and contributing valuable insights into the field of drug stabilization strategies.

## 2. Results and Discussion

### Effect of the Stabilizer Concentrations on the Stability of the Lifitegrast Eyedrop Formulation

Table 2 summarizes the critical factors and responses that influence the stability of the eyedrop formulation, such as appearance, pH, drug content, and impurities. Table 3 presents the corresponding results after storing the eyedrops under accelerated conditions for six months, showing how these stability factors were affected by varying stabilizer concentrations. All eyedrops containing stabilizers met the criteria for each crucial variable throughout the storage period. The formulations maintained a clear appearance at pH 7.2–7.6, and drug content remained consistent within the target range. Impurities remained below 1% in the eyedrops during the 6 months, despite the increase in storage time. In contrast, the eyedrops without citric acid and tromethamine were found to be less stable than the formulations with stabilizers. After six months of storage under accelerated conditions, the drug content in the control samples without stabilizers decreased to 88.2%, and the impurities increased to 1.55%, exceeding the acceptable limits. In addition, the control sample exhibited significant changes in appearance and pH beyond acceptable limits under accelerated conditions. These results indicate that the absence of stabilizers led to significant degradation, demonstrating the crucial role of citric acid and tromethamine in maintaining the physicochemical stability of the formulation.

To better understand these variations in impurities, it is essential to identify the mechanisms by which stabilizers contribute to the physicochemical stability of formulations. The stabilizers used in this study, citric acid and tromethamine, each provide stabilizing effects on lifitegrast through different approaches. Citric acid mainly acts as an antioxidant, effectively preventing oxidative degradation, whereas tromethamine functions as a pH buffer, maintaining a stable pH and preventing the hydrolysis of lifitegrast [12,13,14,15,16]. Although both stabilizers reduced the impurities in the formulations, citric acid demonstrated slightly greater efficacy than tromethamine at equivalent concentrations. Citric acid is known to act as an antioxidant by breaking down ROS such as hydroxyl radicals (OH), superoxide anions (O^2−^), and hydrogen peroxide (H_2_O_2_), and stabilizing them by directly donating hydrogen atoms, thereby inhibiting oxidative damage. It is probable that the antioxidant mechanism efficiently arrested the oxidative degradation of lifitegrast and prevented the formation of impurities [12,13]. Tromethamine maintains the eyedrops at pH 6.8–8.0 as a pH buffer, preventing the hydrolysis of the amide bonds within the lifitegrast structure caused by extreme pH changes [9,10]. In addition, the buffering action of tromethamine reduces its oxidation potential. It can indirectly control the rate of oxidation reactions, which is particularly relevant because oxidation can be accelerated by ROS under acidic conditions, whereas under basic conditions, the non-ionized state of the drug acts as an electron donor, making it more susceptible to oxidation [16].

The physicochemical stability of eyedrops was significantly influenced by the combination and concentration of stabilizers, as demonstrated by the impurities in each formulation. Citric acid and tromethamine each contributed to the stability of lifitegrast through their antioxidant and pH-buffering actions, respectively, but when used together, a synergistic effect on the stabilization of eyedrops was observed via their complementary mechanisms. This synergistic effect can be explained by the following reasons. First, citric acid and tromethamine work together to suppress oxidative degradation and hydrolysis in eyedrops containing both stabilizers, thereby maximizing the stability of lifitegrast. Citric acid mitigates oxidative stress by scavenging ROS, and tromethamine maintains a stable pH to prevent hydrolysis. By inhibiting these two degradation pathways simultaneously, the formulation achieves enhanced stability [9,10]. Second, tromethamine provides a stable pH environment that allows citric acid’s antioxidant function to operate more effectively. The activity of ROS can vary depending on the pH, becoming more reactive in either acidic or alkaline conditions. By maintaining the pH of the eyedrops within the optimal range of pH 6.8–8.0, tromethamine establishes conditions in which citric acid can neutralize ROS effectively, thereby preventing the oxidative degradation of lifitegrast. For this reason, it can be inferred that citric acid and tromethamine complement each other by enhancing both antioxidant protection and pH stability, leading to significantly improved physicochemical stability of the eyedrop formulation [12,13,14,15,16]. While both stabilizers were effective in enhancing the stability of lifitegrast, there was a tendency for more impurities to be generated as the concentrations of citric acid and tromethamine increased. Overuse of these stabilizers can lead to excessive interactions with the drug, reducing the beneficial effects of antioxidant processes or hydrolysis inhibition, and accelerating drug degradation. Therefore, it is essential to use these stabilizers at appropriate concentrations.

**Figure 2:**
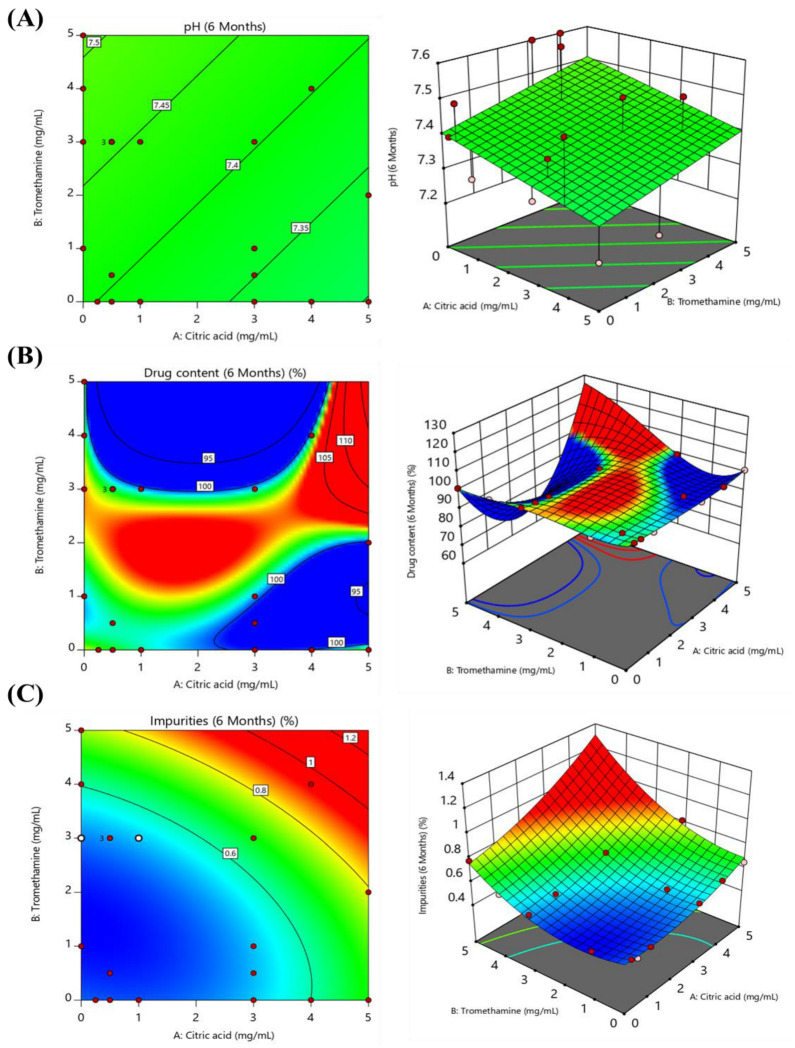
Contour plot and 3D response surface plot showing the effect of stabilizer concentration on (**A**) pH, (**B**) drug content, and (**C**) impurities (the red dots indicate the experimental point).

The effects of the excipients in eyedrops containing lifitegrast were analyzed using statistical methods to determine how the stabilizer concentration affected the response variables. The regression analyses for pH and drug content, presented in Tables S1 and S2, showed that changes in the concentrations of the stabilizer did not significantly impact the response variables. The normal probability plot of the residuals in Figure S1 reveals that the residuals are close to a straight line, suggesting that the model adequately explains the pH and drug content data. However, the actual versus predicted plots for pH and drug content showed poor predictive accuracy for some data. The contour and 3D response surface plots in Figure 2A,B indicate that changes in the concentrations of citric acid and tromethamine had limited effects on pH, whereas for drug content, slight fluctuations were observed at higher concentrations of the stabilizer. The statistical analysis of impurities (Table 4) demonstrated a highly significant model (*p*-value < 0.0001), indicating a considerable effect of the citric acid and tromethamine concentrations. The lack of fit test (*p*-value = 0.146) indicated that the model provided an adequate fit to the data with no significant lack of fit. Figure S1 shows that the residuals closely follow a straight line, and the actual versus predicted plots demonstrate high precision. The contour and 3D response surface plots (Figure 2C) revealed the synergistic effect of the stabilizers in reducing impurities, although an increase in impurities was observed at higher concentrations, emphasizing the importance of selecting appropriate stabilizer levels [21,22,23].

**Figure 3:**
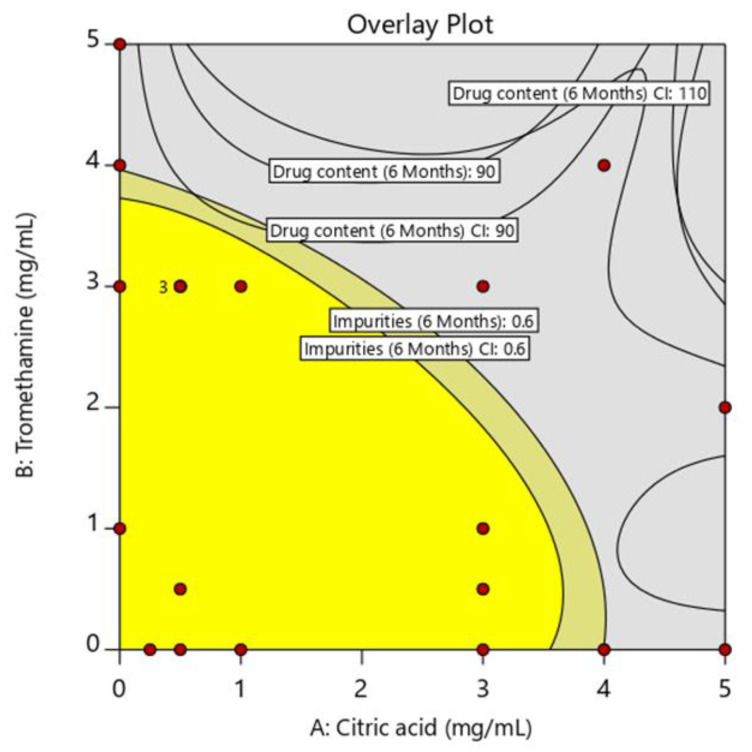
Overlay plot showing the effective stabilizer concentrations for lifitegrast eyedrop formulation (yellow region represents the design space and the red dots indicate the experimental point).

The concentration range of the stabilizers that ensured consistent stability and demonstrated stability of the eyedrops was established using a design space (Figure 3). Drug content and impurities, which varied with stabilizer concentration, were used to establish the design space, as appearance and pH remained stable across formulations and were therefore not considered critical. The acceptable range for drug content was set at 90–110%, and the impurity threshold was set at 0.6% to ensure excellent stability and robustness of the formulation. The yellow area represents the concentration range of stabilizers in which both the drug content and impurity criteria are met, ensuring long-term product stability. The experimental results and statistical analyses demonstrated that as the concentrations of citric acid and tromethamine decreased, the number of impurities also decreased. Therefore, the concentration of the stabilizer was set at a maximum of 2.5 mg/mL for citric acid and 2 mg/mL for tromethamine, which is expected to ensure sufficient stability of the eyedrops and maintain high product quality [24,25,26].

## 3. Materials and Methods

### 3.1. Materials

Lifitegrast (99.5% purity, as is) was purchased from MSN Life Sciences Pvt., Ltd. (Chandampet, Telangana, India). Tromethamine (99.6% purity, as is) was purchased from Merck KGaA (Darmstadt, Germany). Anhydrous citric acid (99.5% purity), anhydrous sodium phosphate dibasic (99% purity), sodium chloride (99.5% purity), 35% hydrochloric acid, sodium hydroxide (97% purity), and 70% perchloric acid were purchased from Daejung Chemicals & Metals Co., Ltd. (Siheung, Republic of Korea). Acetonitrile (HPLC-grade, ≥99.9%) was purchased from Honeywell Burdick & Jackson (Morristown, NJ, USA). Ultrapure water was supplied using a Millipore Milli-Q purification system (Molsheim, France). All other chemicals were of analytical grade and used as received without further purification.

### 3.2. Preparation of Lifitegrast Eyedrop Formulations

To evaluate the effect of citric acid and tromethamine concentrations on the stability of lifitegrast eyedrops, the formulations were prepared with a lifitegrast concentration of 5%, consistent with the commercially available Xiidra^®^ ophthalmic solution (Novartis Pharma AG, Basel, Switzerland). Because the formulation was intended for ophthalmic use, excipients were added to ensure proper buffering capacity, osmotic pressure, and pH control. Anhydrous sodium phosphate (3.55 mg/mL) was first dissolved in purified water to act as a buffer, followed by the addition of sodium chloride to adjust the osmolarity to 230–320 mOsmol/kg. Sodium chloride, a common excipient in ophthalmic solutions, was added as required to mimic the osmotic pressure of tears and minimize ocular irritation. The osmolarity of the eyedrop formulations was measured using an osmometer (Osmomat 3000; Gonotec GmbH, Berlin, Germany). Citric acid and tromethamine were then added, and lifitegrast was dispersed in the solution. Because the drug is more soluble under basic conditions, the pH was gradually increased by adding small amounts of 1 M NaOH to facilitate its dissolution. Once the lifitegrast was fully dissolved, the final solution was adjusted to pH 7.4 using 1 M NaOH or 1 M HCl, as needed, to achieve the target pH [27,28]. To determine the detailed stabilizer concentrations in formulations, an experimental design matrix was constructed using the Design Expert^®^ 11.0 software (Stat-Ease, Inc., Minneapolis, MN, USA). The independent variables were concentrations of citric acid (X_1_) and tromethamine (X_2_), each ranging from 0 to 5 mg/mL (Table 2). Twenty experimental points, including three replicates, were designed, as presented in Table 3.

### 3.3. Evaluation of Formulation Characteristics

To evaluate the effect of stabilizers on the formulations, the prepared lifitegrast eyedrops were stored in a temperature and humidity chamber (TH3-KE-025; Jeio Tech Co. Ltd., Daejeon, Republic of Korea) under the accelerated conditions of 40 °C ± 2 °C and 75% relative humidity, in accordance with ICH guidelines. The critical stability indicators—appearance, pH, drug content (%), and impurities (%)—were evaluated at the initial time point, as well as at 1, 3, and 6 months, serving as critical response variables for assessing the stability of lifitegrast eyedrops [21,29,30,31]. The criteria for each of these response variables were established according to ICH guidelines Q1A, Q6A, and Q3B, as shown in Table 2 [22,23]. The appearance of the formulation was assessed by visual inspection of the changes in color and transparency. Additionally, the occurrence of precipitation was monitored to evaluate the physical stability of the formulation. The pH of the formulations was measured using a pH meter (SevenCompact S210; Mettler Toledo, Greifensee, Switzerland). The drug content of the eyedrops was analyzed using an ultra-high-performance liquid chromatography (UHPLC) system (Nexera X3; Shimadzu, Tokyo, Japan). The sample was prepared by diluting the eyedrops with methanol to obtain the lifitegrast concentration of 100 μg/mL. The standard solution was then diluted with methanol to obtain the same concentration. Both the standard and sample solutions were injected into the system at 5 μL each and separated on a Gemini C18 reversed-phase column (150 mm × 4.6 mm, 5 µm; Phenomenex, Torrance, CA, USA) maintained at 30 °C. The mobile phase, consisting of 0.2% perchloric acid and acetonitrile (51:49, *v*/*v*), was delivered at a flow rate of 0.8 mL/min, and detection was performed at 260 nm. The lifitegrast content (%) was calculated based on the ratio of the measured to the theoretical concentration, as shown in Equation (1).

(1) $$Drug\;content\left(\%\right)=\frac{Measured\;lifitegrast\;concentration\;(mg/mL)}{Theoretical\;lifitegrast\;concentration\;(mg/mL)}\times 100$$

Impurities in the eyedrops were analyzed using a HPLC system (Agilent 1260 Infinity; Agilent Technologies, Santa Clara, CA, USA) (Figure S2). The standard and sample solutions were prepared by diluting with a 1:1 mixture of acetonitrile and methanol as the diluent to achieve lifitegrast concentrations of 1 μg/mL and 1 mg/mL, respectively. The mobile phase consisted of perchloric acid (A) and a perchloric acid–acetonitrile mixture (30:70, *v*/*v*) (B) and was used in the gradient mode, as detailed in Table 5, with a flow rate of 1.0 mL/min. The analysis was conducted using a Gemini NX-C18 column (250 mm × 4.6 mm, 3 µm, Phenomenex, Torrance, CA, USA) at 30 °C. The system was injected with 5 µL of the sample, maintained at 5 °C, with detection performed at 215 nm. The amounts of individual impurities in the eyedrops were calculated using Equation (2), and the total impurities were determined by summing the individual amounts.

(2) $$Impurity\left(\%\right)=\frac{SAM\;A}{STD\;A}\times \frac{SAM\;V}{SAM\;M}\times C\times RF\times 100$$

SAM A: peak area of the individual impurity in the sample solution;

STD A: average peak area of lifitegrast in the standard solution;

SAM V: dilution volume of the sample (mL);

SAM M: weight of lifitegrast in the sample (mg);

C: concentration of lifitegrast in the sample (mg/mL);

RF: response factor for the individual impurity;

100: conversion factor to express the value as a percentage.

## 4. Conclusions

In this study, citric acid and tromethamine were used to enhance the physicochemical stability of eyedrops containing lifitegrast. The effects of stabilizer concentration on critical parameters such as appearance, pH, drug content, and impurities were thoroughly evaluated to ensure formulation stability. Owing to the high sensitivity of the drug to oxidative stress and pH changes, a strategy was implemented that utilizes the antioxidant properties of citric acid and the buffering action of tromethamine to prevent both oxidation and hydrolysis. The results showed that citric acid and tromethamine were effective in maintaining the appearance, pH, and drug content within acceptable limits across all eyedrops. However, an increase in impurities was observed with higher stabilizer concentrations, indicating the need for precise control of stabilizer levels to prevent degradation. An appropriate concentration range for citric acid and tromethamine was determined through the analysis of response factors such as appearance, pH, content, and impurities, effectively minimizing impurities and ensuring the physicochemical stability of the eyedrops. The formulation is expected to maintain long-term stability and comply with the quality requirements within the concentration limits defined by the design space. These findings provide valuable insights for the development of more stable lifitegrast eyedrops and may serve as a reference for future commercial production and clinical applications, ensuring both stability and efficacy over time.

## Figures and Tables

**Table 1:** Quality target product profile (QTPP) for lifitegrast eyedrops.

QTPP Elements	Target	Justification
Dosage form	Ophthalmic solution	Common dosage form used for treating dry eye disease.
Route of administration	Ocular route	Suitable route for treating inflammation on the ocular surface, the primary cause of dry eye disease.
Efficacy	Treatment of dry eye disease	Lifitegrast treats the root cause of multifactorial dry eye disease, providing anti-inflammatory effects on the cornea and conjunctiva, improving symptoms [4,5].
Dosage strength	Twice daily, both eyes, 5% lifitegrast	Consistent with Xiidra^®^ ophthalmic solution, ensuring therapeutic efficacy and safety [6].
Stability	Stable for at least 6 months under accelerated conditions (40 °C ± 2 °C, 75% relative humidity)	Ensures stability in terms of appearance and drug content, preventing degradation during long-term storage.

**Table 2:** Factors and responses for eyedrop stability.

Factors	Level	
	Low (−1)	High (+1)
X_1_: Concentration of citric acid (mg/mL)	0	5
X_2_: Concentration of tromethamine (mg/mL)	0	5
Responses	Goal	
Y_1_: Appearance	Clear solution	
Y_2_: pH	6.8–8.0	
Y_3_: Drug content (%)	90.0–110.0	
Y_4_: Impurities (%)	<1.0	

**Table 3:** Evaluation of the eyedrops formulation manufactured according to the experimental design matrix.

Run	Factors		Responses															
	X_1_ Conc. of Citric Acid (mg/mL)	X_2_ Conc. of Tromethanine (mg/mL)	Y_1_ Appearance				Y_2_ pH				Y_3_ Drug Content (%)				Y_4_ Impurities (%)			
			Time (Months)				Time (Months)				Time (Months)				Time (Months)			
			0	1	3	6	0	1	3	6	0	1	3	6	0	1	3	6
1	5	0	Clear	Clear	Clear	Clear	7.5	7.4	7.5	7.2	100.21	100.32	100.70	101.74	0.23	0.41	0.58	0.65
2	0	5	Clear	Clear	Clear	Clear	7.4	7.3	7.3	7.4	100.43	100.21	100.53	101.41	0.24	0.43	0.63	0.78
3	5	2	Clear	Clear	Clear	Clear	7.4	7.4	7.5	7.2	101.12	100.92	101.33	99.82	0.24	0.46	0.68	0.82
4	0.5	0	Clear	Clear	Clear	Clear	7.5	7.4	7.4	7.5	100.92	100.53	100.24	101.42	0.21	0.37	0.44	0.46
5	3	0.5	Clear	Clear	Clear	Clear	7.5	7.4	7.3	7.4	100.54	100.72	100.74	99.84	0.23	0.32	0.41	0.49
6	1	3	Clear	Clear	Clear	Clear	7.3	7.3	7.3	7.6	99.72	99.93	99.82	100.60	0.23	0.41	0.52	0.58
7	0.5	0.5	Clear	Clear	Clear	Clear	7.4	7.4	7.3	7.4	101.23	100.83	101.31	101.23	0.22	0.31	0.39	0.44
8	1	0	Clear	Clear	Clear	Clear	7.4	7.4	7.4	7.3	99.83	100.64	100.51	100.50	0.22	0.38	0.46	0.48
9	0	3	Clear	Clear	Clear	Clear	7.5	7.4	7.5	7.6	99.64	99.90	99.90	102.82	0.24	0.37	0.47	0.54
10	3	0	Clear	Clear	Clear	Clear	7.4	7.3	7.3	7.3	100.60	100.52	100.42	99.84	0.22	0.38	0.49	0.56
11	4	4	Clear	Clear	Clear	Clear	7.4	7.4	7.4	7.5	100.82	100.41	100.93	100.62	0.23	0.48	0.72	0.92
12	0	4	Clear	Clear	Clear	Clear	7.5	7.5	7.5	7.6	100.53	100.93	100.54	101.20	0.24	0.38	0.52	0.60
13	0	1	Clear	Clear	Clear	Clear	7.4	7.4	7.5	7.4	100.94	101.14	101.22	100.11	0.20	0.35	0.42	0.47
14	3	1	Clear	Clear	Clear	Clear	7.3	7.2	7.5	7.3	99.82	99.70	100.31	99.93	0.23	0.37	0.48	0.57
15	0.25	0	Clear	Clear	Clear	Clear	7.5	7.4	7.5	7.4	101.21	101.43	101.63	101.62	0.21	0.37	0.43	0.49
16	4	0	Clear	Clear	Clear	Clear	7.4	7.4	7.4	7.5	99.70	99.92	99.83	100.14	0.22	0.39	0.55	0.62
17	3	3	Clear	Clear	Clear	Clear	7.3	7.4	7.4	7.5	100.11	100.44	100.62	100.62	0.24	0.43	0.57	0.68
18	0.5	3	Clear	Clear	Clear	Clear	7.5	7.6	7.5	7.2	99.62	99.71	99.74	100.53	0.22	0.37	0.47	0.47
19	0.5	3	Clear	Clear	Clear	Clear	7.3	7.4	7.3	7.4	99.82	99.93	100.11	101.43	0.23	0.38	0.46	0.49
20	0.5	3	Clear	Clear	Clear	Clear	7.4	7.4	7.5	7.4	100.13	100.12	100.33	100.91	0.24	0.39	0.45	0.46

**Table 4:** Summary of model fitting and statistical analysis for Y_4_ (impurities, %).

Source	Sum of Squares	Degree of Freedom	Mean Square		F-Value	*p*-Value	
Model	0.3211	5	0.0642		50.01	<0.0001	*significant*
X_1_	0.2099	1	0.2099		163.50	<0.0001	
X_2_	0.1681	1	0.1681		130.89	<0.0001	
X_1_X_2_	0.0245	1	0.0245		19.08	0.0006	
X_1_^2^	0.0114	1	0.0114		8.86	0.0100	
X_2_^2^	0.0454	1	0.0454		35.37	<0.0001	
Residual	0.0180	14	0.0013				
Lack of Fit	0.0175	12	0.0015		6.25	0.1460	*not significant*
Pure Error	0.0005	2	0.0002				
Cor Total	0.3391	19					
Standard Error: 0.0358				Adjusted R-Squared: 0.9280			
R-Squared: 0.9470				Prediction R-Squared: 0.8604			
Regression Equation of the Fitted Model							
Y_4_ = 0.4854 − 0.0195 X_1_ − 0.0685 X_2_ + 0.0139 X_1_X_2_ + 0.0121 X_1_^2^ + 0.0246 X_2_^2^							

**Table 5:** Gradient HPLC method for impurities analysis of eyedrops.

Time (min)	Mobile Phase A (%)	Mobile Phase B (%)
0.01	55	45
3	55	45
18	40	60
37	8	92
50	8	92
51	55	45
60	55	45

Note: Mobile phase A consisted of perchloric acid buffer, whereas mobile phase B was a mixture of perchloric acid buffer and acetonitrile (30:70, *v*/*v*).

## Data Availability

Data are contained within the article.

## Supplementary Materials

The following supporting information can be downloaded at: https://www.mdpi.com/article/10.3390/ph17111415/s1, Figure S1: Normal probability plots and linear correlation plots between the actual and predicted values of (A) pH, (B) content, and (C) impurities; Figure S2: HPLC chromatogram showing the separation of degradation products; Table S1: Summary of model fitting and statistical analysis for Y2 (pH); Table S2: Summary of model fitting and statistical analysis for Y3 (content, %).
