# Supplementary material for: Effect of Citric Acid and Tromethamine on the Stability of Eyedrops Containing Lifitegrast
Source: Pharmaceuticals (Basel). 2024 Oct 23;17(11):1415. doi: 10.3390/ph17111415 (PMC11597827; doi:10.3390/ph17111415)
Supplement: Supplementary file 1 [file pharmaceuticals-17-01415-s001.zip › pharmaceuticals-3250226-supplementary.pdf]

Supplementary materials

Effect of Citric Acid and Tromethamine on the Stability of Eyedrops Containing Lifitegrast

Ji-Su Jeong [1,†], Eun-Sol Ha [1,†], Heejun Park [2], Seon-Kwang Lee [1], Hui-Taek Kang [1], Min-Soo Kim [1,*]

[1] College of Pharmacy, Pusan National University, 63 Busandaehak-ro, Geumjeong-gu, Busan 46241, Republic of Korea; sui15@pusan.ac.kr (J.-S.J); edel@pusan.ac.kr (E.-S.H.); lsk7079@pusan.ac.kr (S.-K.L); gms1406@pusan.ac.kr (H.-T.K.)
[2] College of Pharmacy, Duksung Women's University, 33, Samyangro 144-gil, Dobong-gu, Seoul 01369, Republic of Korea; heejunpark@duksung.ac.kr
* Correspondence: minsookim@pusan.ac.kr; Tel.: +82-51-510-2813
† These authors contributed equally to this work

Validation for the used analytical method

The validation process followed the guidelines set by the International Council for Harmonisation (ICH) Q2(R1), which emphasizes crucial criteria such as linearity, accuracy, precision, and specificity. For linearity, the method demonstrated excellent correlation across a concentration range from 0.1 μg/mL to 100 μg/mL, with a determination coefficient (R^2) consistently above 0.999, confirming the method's robustness over a wide range of concentrations. Precision was assessed by calculating the relative standard deviation (RSD) for analyses, and the RSD was less than 2%, indicating high reproducibility. Accuracy was verified by comparing the measured values to known concentrations, with recovery rates consistently falling within the 95-105% range, thus ensuring that the method provides reliable and accurate results. In terms of specificity, we confirmed that lifitegrast could be accurately detected without interference from other components or degradation products. Furthermore, certified reference standards for lifitegrast were used to ensure the reliability of the analysis, thus reinforcing the validity of the stability testing results.

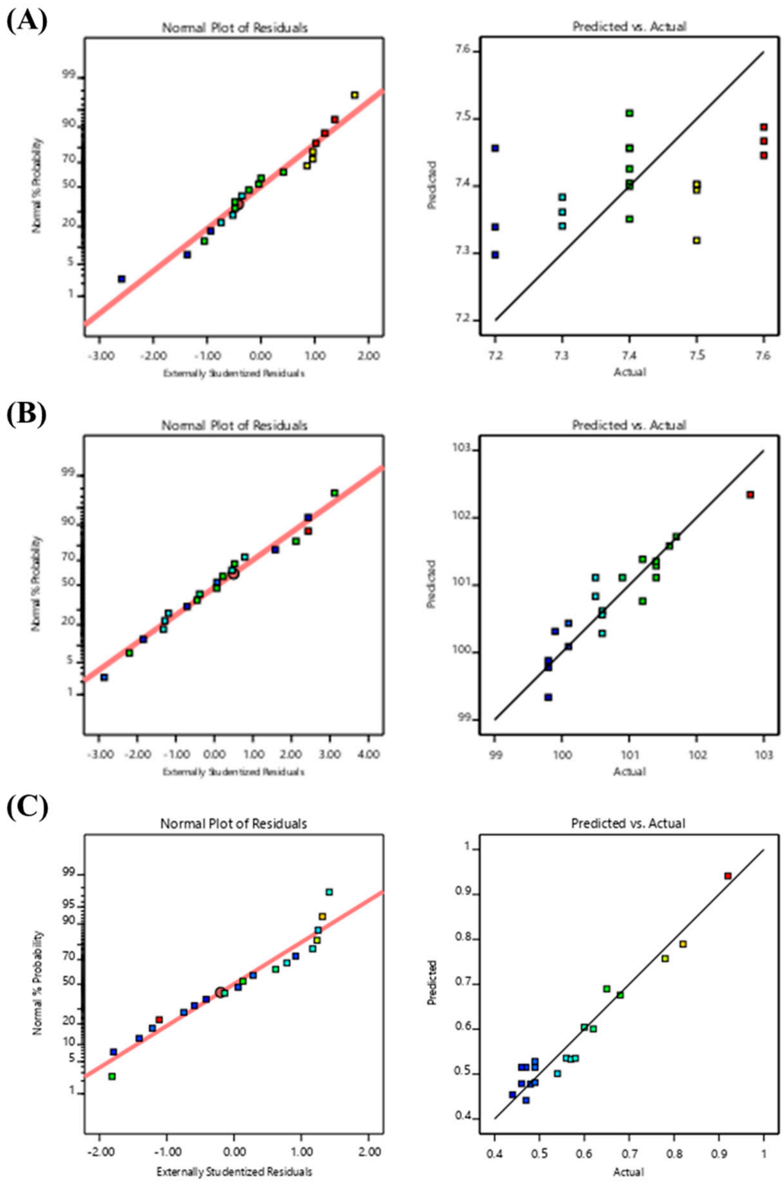

Figure S1. Normal probability plots and linear correlation plots between the actual and predicted values of (A) pH, (B) content and (C) impurities.

HPLC chromatogram showing the separation of degradation products

It was verified that the method used for analyzing the chemical stability of lifitegrast has been designed to effectively separate lifitegrast from its degradation products. The chromatogram provided in the response clearly illustrates the separation of the lifitegrast peak from various degradation products observed in samples stored under accelerated stability conditions. In the chromatogram, the upper red line represents the chromatogram of the lifitegrast-containing eyedrop sample stored under stress, where distinct peaks corresponding to different degradation products can be seen. For comparison, the blue line represents a blank sample without any lifitegrast, showing no interfering peaks, thus confirming the specificity of our analytical method. The method's ability to distinguish lifitegrast from its degradation products was confirmed using an analysis procedure obtained from the lifitegrast manufacturer, which outlines specific conditions to achieve clear peak separation. This method follows established procedures for separating lifitegrast and degradation products under stress conditions. It has been effectively used to identify and quantify lifitegrast and its degradation products, ensuring accurate stability analysis.

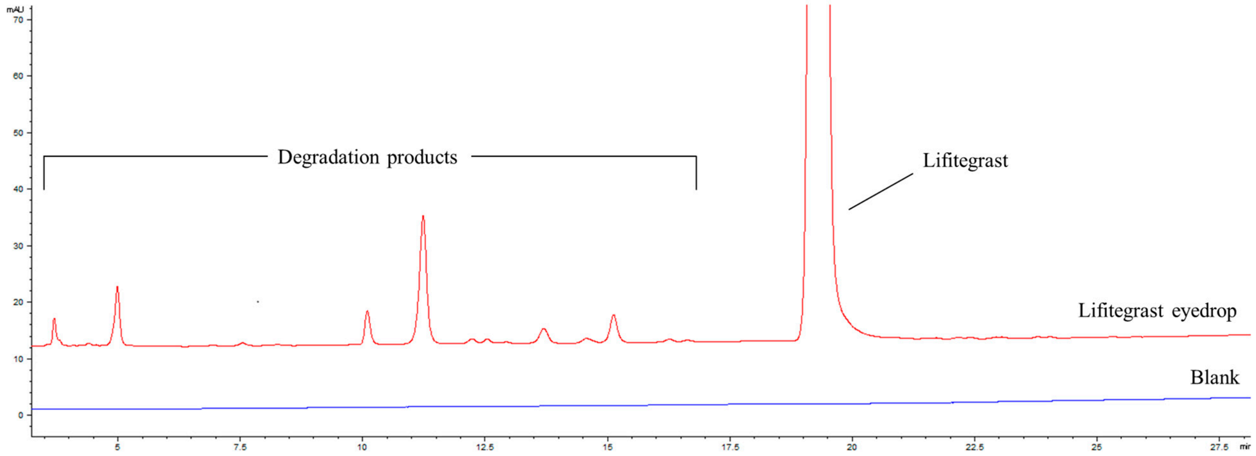

Figure S2. HPLC chromatogram showing the separation of degradation products.

Table S1. Summary of model fitting and statistical analysis for Y_2 (pH).

Source	Sum of Squares	Degree of Freedom	Mean Square	F-value	*p*-value	
Model	0.0642	2	0.0321	2.23	0.138	*not significant*
X_1	0.0260	1	0.0260	1.80	0.197	
X_2	0.0210	1	0.0210	1.45	0.244	
Residual	0.2453	17	0.0144			
Lack of Fit	0.2186	15	0.0146	1.09	0.578	*not significant*
Pure Error	0.0267	2	0.0133			
Cor Total	0.3095	19				

Standard Error: 0.1201	Adjusted R-squared: 0.1143
R-Squared: 0.2086	Prediction R-squared: -0.1053

Regression equation of the fitted model

$$Y_2 = 7.4050 - 0.0214\ X_1 - 0.0207\ X_2$$

Table S2. Summary of model fitting and statistical analysis for Y_3 (Content, %).

Source	Sum of Squares	Degree of Freedom	Mean Square	F-value	*p*-value	
Model	10.2787	14	0.7342	2.20	0.1971	*not significant*
X_1	0.0064	1	0.0064	0.02	0.8955	
X_2	0.5380	1	0.5380	1.61	0.2603	
X_1X_2	0.4930	1	0.4930	1.48	0.2787	
X_1^2	0.0001	1	0.0001	0.01	0.9934	
X_2^2	0.5928	1	0.5928	1.77	0.2404	
$X_1^2X_2$	0.3858	1	0.3858	1.15	0.3317	
$X_1 X_2^2$	0.8622	1	0.8622	2.58	0.1691	
X_1^3	0.0377	1	0.0377	0.11	0.7507	
X_2^3	0.2249	1	0.2249	0.67	0.4493	
$X_1^2 X_2^2$	0.4588	1	0.4588	1.37	0.2941	
$X_1^3X_2$	0.0054	1	0.0054	0.02	0.9041	
$X_1 X_2^3$	0.1518	1	0.1518	0.45	0.5302	
X_1^4	0.0295	1	0.0295	0.09	0.7784	
X_2^4	1.2809	1	1.2809	3.83	0.1076	
Residual	1.6708	5	0.3342			
Lack of Fit	1.2641	3	0.4214	2.07	0.3419	*not significant*
Pure Error	0.4067	2	0.2033			
Cor Total	11.9495	19				

Standard Error: 0.5781

Adjusted R-squared: 0.4687

R-Squared: 0.8602

Prediction R-squared: -304.9347

Regression equation of the fitted model

$$Y_3 = 101.9402 – 1.5772 X_1 – 4.9538 X_2 + 5.1650 X_1X_2 + 0.6279 X_1^2 + 4.7085 X_2^2 – 1.5987 X_1^2 X_2 - 1.4238 X_1X_2^2 – 0.1795 X_1^3 - 1.3880 X_2^3 + 0.5703 X_1^2X_2^2 + 0.0155 X_1^3X_2 – 0.1488 X_1X_2^3 + 0.0231 X_1^4 + 0.1280 X_2^4$$
